# Thiolane-type sulfides from garlic, onion, and Welsh onion

**DOI:** 10.1007/s11418-021-01533-x

**Published:** 2021-06-03

**Authors:** Toshihiro Nohara, Yukio Fujiwara, Mona El-Aasr, Tsuyoshi Ikeda, Masateru Ono, Daisuke Nakano, Junei Kinjo

**Affiliations:** 1 Faculty of Pharmaceutical Sciences, Sojo University, 4-22-1, Ikeda, Nishi-ku, Kumamoto, 860-0082 Japan; 2 Graduate School of Medical Sciences, Faculty of Life Sciences, Kumamoto University, 1-1-1, Honjo, Chuo-ku, Kumamoto, 860-8556 Japan; 3 Faculty of Pharmacy, Tanta University, Tanta, 31111 Egypt; 4 School of Agriculture, Tokai University, 9-1-1, Toroku, Higashi-ku, Kumamoto, 862-8652 Japan; 5 Faculty of Pharmaceutical Sciences, Fukuoka University, 8-19-1, Nanakuma, Jonan-ku, Fukuoka, 814-0180 Japan

**Keywords:** Garlic, Onion, Welsh onion, 3,4-dimethylthiolane-type, Onionin A_1_, Garlicnin B_1_, Antitumor effect

## Abstract

In this paper, we review our work in the last 10 years wherein we examined the sulfides in the acetone extracts of garlic (*Allium sativum*), onion (*A. cepa*), and Welsh onion (*A. fistulosum*), obtained and characterized the structures of new sulfides, three 3,4-dimethylthiolane-type sulfides from onion and Welsh onion, respectively, and four acyclic-type, nine 3,4-dimethyl- thiolane-type, four 2-methylthiolane (and thiane)-type, two 1,2-dithiolane-type, and two 2-oxothiolane-type sulfides, together with (*E*)-ajoene and one kujounin-type sulfide from garlic. During this process, structural corrections were made in onionin A group, garlicnin A, and garlicnin B group in some 3,4-dimethylthiolane-type sulfides. Next, hypothetical pathways for the production of the aforementioned sulfides were proposed. Furthermore, it was revealed that a typical 3,4-dimethylthiolane-type sulfide, onionin A_1_ obtained from onion, having the isomeric structure of garlicnin B_1_ obtained from garlic, decreased tumor proliferation and controlled tumor metastasis. These results showed that onionin A_1_ is an effective agent for controlling tumors, and that the antitumor effects observed in vivo are likely caused by reversing the antitumor immune system. Activation of the antitumor immune system by onionin A_1_ might be an effective adjuvant therapy for patients with osteosarcoma, ovarian cancer and other malignant tumors.

## Introduction

Garlic (*Allium sativum* L.) is ranked at the top of the list of designer foods showing anti-cancer effects by the National Cancer Institute [1]. Generally, the biological activity of garlic is distinguished in two categories: cardiovascular disease prevention and cancer prevention. Activities in the former category include the inhibition of cholesterol synthesis, platelet aggregation, and arterial smooth muscle cell proliferation, as well as anti-inflammatory, antioxidant, and hydrogen sulfide-mediated vasodilatory effects. The activities in the latter category include the effects on carcinogen metabolism, *i.e.*, enhanced cellular glutathione synthesis that induces cell cycle arrest and apoptosis, and prevention of *Helicobacter pylori* infection, gastric cancer, and colorectal cancer [2–6].

The chemistry of *Allium* sulfides began with the discovery of allicin and alliin in 1944 [7] and 1951 [8], respectively, in garlic. In 1971, two types of vinyldithiin derivatives [9] were identified as thermally decomposed compounds by GC analysis of allicin. In 1984, Block and Ahmad determined the structure of ajoene in ether fraction [10]. It was also found that volatile garlic oils contained many sulfur compounds, such as diallylsulfide, (*Z* and *E*)-ajoene, 1,3-vinyldithiin, and 1,2-vinyldithiin, produced by the decomposition of thiosulfinates [11]. Unexpectedly, there were few clarified sulfides from garlic; in particular, cyclic sulfides before our study. Therefore, we had started the investigation for aiming at the isolation, structural characterization, and antitumor activity of the cyclic sulfides (sulfur-containing compounds including sulfoxides) from garlic, onion (*A. cepa*), and Welsh onion (*A. fistulosum*). The present review provides a brief description of the above-mentioned study.

## Extraction and separation of garlic

Acetone was selected as the extracting solvent because it was expected to prolong the lifetime of allyl (or 1-propenyl) sulfenic acid and allyl thiosulfenic acid, which are derived easily by the decomposition of allicin. The acyclic and cyclic sulfides are stabilized by the electron-inductive interaction between acetone and sulfenic acids, and between acetone and cyclic sulfide. Chinese garlic was used, which is the same as Japanese garlic, because it was readily available and the occurrence of various sulfides, due to long drying storage, was expected. Chinese garlic (1.0 kg) was chopped and blended with acetone in a mixer. The mixtures were then soaked in acetone for 3 days at room temperature. During this time, sulfenic acid analogs might undergo chemical changes, such as cyclization and artificial reactions, to produce new sulfides. In particular, we intended to obtain stable cyclic sulfides possessing antitumor activity. Next, the filtrate was concentrated at 40 °C in vacuum to obtain the extract in a small volume that was partitioned between ethyl acetate and water. The ethyl acetate extractive (5.9 g) was separated by column chromatography on silica gel eluting with *n*-hexane: acetone (from 6: 1 to 2: 1) to yield 21 new sulfides named garlicnins A (48.2 mg) [12], B_1_ (242.0 mg), B_2_ (47.2 mg), B_3_ (29.8 mg), B_4_ (19.3 mg), C_1_ (26.4 mg), C_2_ (23.4 mg), C_3_ (14.6 mg) [13, 14], G (17.2 mg), I_1_ (17.4 mg) [15], I_2_ (15.6 mg) [16], J_1_ (17.4 mg) [15], J_2_ (19.4 mg) [17], L-1 (47.2 mg), L-2 (19.8 mg), L-3 (19.3 mg), L-4 (23.4 mg) [18], M (21.1 mg) [16], P (18.4 mg) [17], and onionins B_1_ (27.4 mg), and B_2_ (26.2 mg) [19], together with the known sulfide, (*E*)-ajoene (279.7 mg) [10], and kujounin A_1_ derivative (22.1 mg), which related to kujounin A_1_ obtained from *Allium fistulosum* by Matsuda et al*.* [20]. The structures of the obtained sulfides were characterized using high-resolution fast atom bombardment mass spectroscopy (HR-FABMS), ^1^H-NMR, ^13^C-NMR, ^1^H-^1^H NMR correlation spectroscopy (COSY), ^1^H-detected heteronuclear correlation through multiplet quantum coherence (HMQC), heteronuclear multiple bond correlation (HMBC) and nuclear Overhauser effect spectroscopy (NOESY). To determine the relative steric configuration of the cyclic sulfides, aromatic solvent-induced NMR shifts were applied [21, 22].

## Extraction and separation of onion and Welsh onion

Similarly, the extraction and separation of onion (*A. cepa*) and Welsh onion (*A. fistulosum*) were performed. From onion bulbs (640 g), onionin A_1_ (42.2 mg) [23], onionin A_2_ (23.5 mg), onionin A_3_ (16.2 mg) [24], onionin B_1_ (16.4 mg), and B_2_ (20.5 mg) [19], were obtained, and from Welsh onion leaves (1.1 kg), onionin A_1_ (34.2 mg), onionin A_2_ (22.1 mg) and onionin A_3_ (16.4 mg) [24] were obtained.

## Structures of isolated sulfides from garlic, onion, and Welsh onion

**Fig. 1:**
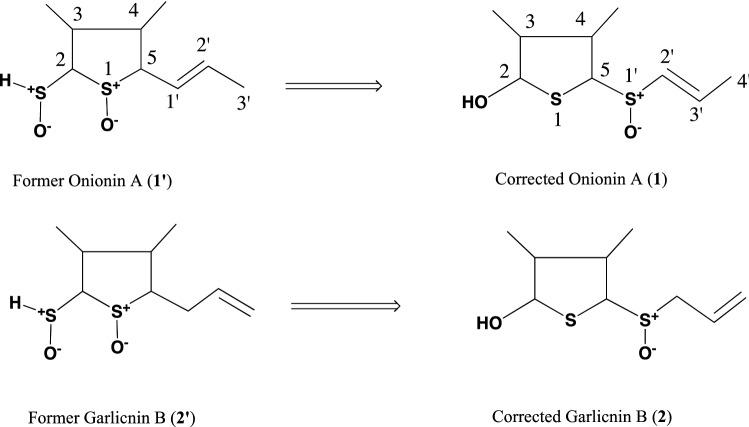
Corrected structures of onionin A_1_ (**1**) and garlicnin B_1_ (**2**)

**Fig. 2:**
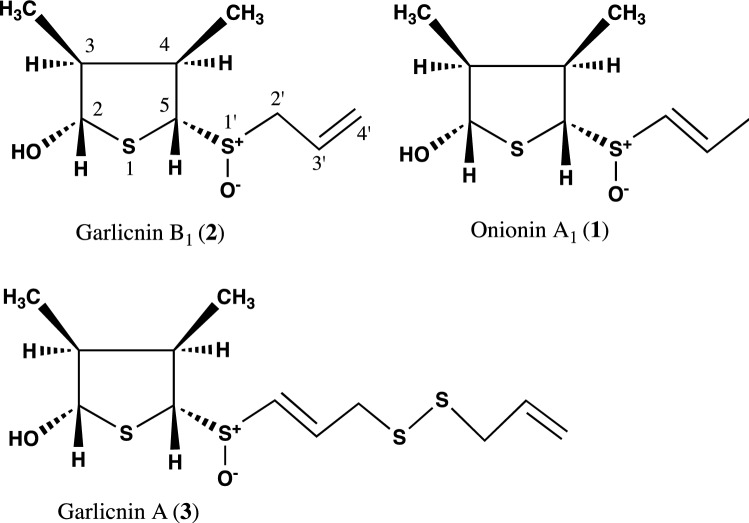
Structures of onionin A_1_ (**1**), garlicnin B_1_ (**2**), and garlicnin A (**3**)

The above garlicnins and onionins were divided into five types: acyclic-type sulfides including garlicnins L-1, L-2, L-3, and L-4; major sulfides, 3,4-dimethylthiolane-type sulfides including garlicnins A, B_1_, B_2_, B_3_, B_4_, C_1_, C_2_, C_3_, and M, onionins A_1_, A_2_, and A_3_; 2-methylthiolane (and thiane)-type sulfoxides including garlicnins I_1_, I_2_, J_1_ and J_2_; 1,2-dithiolane-type sulfoxides including garlicnins G and P; and 2-oxothiolane-type sulfides including onionins B_1_ and B_2_. The structures of acyclic-type sulfides, that is, garlicnins L-1, L-2, L-3, and L-4, were characterized as *E*-5-thiaocta-6-ene 4-methyl-2,5-dioxide, *E*-2,6,7-trithiadeca-4,9-diene 2-oxide, *Z*-4,5,9,10-tetrathiatrideca-1,7,12-triene, and *E*-6,7-dithiadeca-2.9-diene 2-methyl-1-oxide, respectively. Regarding the 3,4-dimethylthiolane-type sulfides, we determined the structure of onionin A_1_, prior to the structure determination of garlicnin B group from garlic, as 3,4-dimethylthiolane *S*-oxide (**1’**) in 2010 as shown Fig. 1, based on the ^1^H-^1^H COSY analysis that included the correlation between H-5 and H-1’, the proton assignments of H–S^+^-O^−^ and H-2 at C-2, and determination of the relative configuration by the aromatic solvent-induced NMR shifts [21, 22]. In relation to the structure of onionin A_1_, we determined the structure of garlicnin B_1_ (**2’**) isolated from garlicin in 2012. However, in 2018, Block et al. corrected the structure of garlicnin B_1_ as 3,4-dimethyl-5-allylsulfinylthiolane- 2-ol (**2**) [25] as shown in Fig. 1. This correction was made because the proposed continuity of nine carbons was not observed in the ^13^C-^13^C NMR incredible natural abundance double quantum transfer experiments (INADEQUATE). In 2019, Kubec et al. corrected onionin A_1_ as (*E*)-3,4-dimethyl-5-(1-propenylsulfinyl)thiolane-2-ol (**1**) as shown in Fig. 1, and he only corrected the part of structure and retained the names onionin A and garlicnin B [26]. Here, we reconfirmed the validity of their claims and we reformed the structures of onionin A_1_ (**1**) and garlicnins B_1_ (**2**), and determined the absolute configuration [27] of garlicnin B_1_ as shown in Fig. 2 by the Mosher method [28, 29] and NOESY analysis of **2**. Simultaneously, the absolute configurations of onionin A_1_ and garlicnin A (**3**) were also deduced because their proton chemical shifts of H-2, H-3, H-4, CH_3_ at C-3, and CH_3_ at C-4, and their carbon chemical shifts of C-2, C-3, C-4, CH_3_ at C-3, and CH_3_ at C-4 approximated to those of garlicnin B_1_ (**2**) as shown in Fig. 2.

**Fig. 3:**
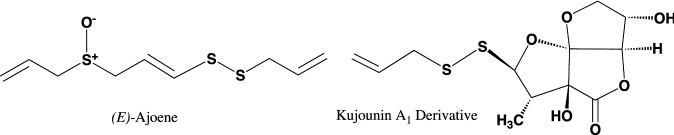
Structures of (*E*)-ajoene and kujounin A_1_ derivative

The structures of garlicnins C_1_, C_2_, and C_3_ were determined to be 2-(allyldisulfanyl)-5-(1-propenylsulfinyl)-3,4-dimethylthiolan-*S*-oxide. Garlicnins C_1_, C_2_, and C_3_ are steric isomers. The structure of garlicnin M was determined to be 2,5-bis(allyldisulfanyl)-3,4-dimethyl-thiolane-*S*-oxide. Next, the structures of 2-methylthiolane-type sulfoxides, that is, garlicnins I_1_ and I_2_ were determined to be 5-methyl-2-(allyldisulfanyl)-3-[(allyldisulfanyl)-methyl]-thiolane-*S*-oxides, and the structures of 2-methylthiane-type sulfoxides; garlicnins of J_1_ and J_2_ were determined to be 6-methyl-2,3-bis(allyldisulfanyl)-thiane-*S*-oxide and 6-methyl-4-(allyl- disulfanyl)-thiane-*S*-oxide, respectively. The structures of 1,2-dithiolane-type sulfoxides; garlicnins G and P were determined to be 4-(allyl)-3-(allylsulfinyl)-1,2-dithiolane, and 3-methyl-2,7,8-trithia-bicyclo[3.3.0]octan-2-oxide, respectively. Finally, 2-oxothiolane-type sulfides, onionins B_1_ and B_2_ were determined to be 5-(allyldisulfanyl)-3,4-dimethyl-2-oxothiolanes. The structures of the above garlicnins and onionins are summarized in Table 1, together with (*E*)-ajoene and kujounin A_1_ derivative as shown in Fig. 3.

**Table 1:**
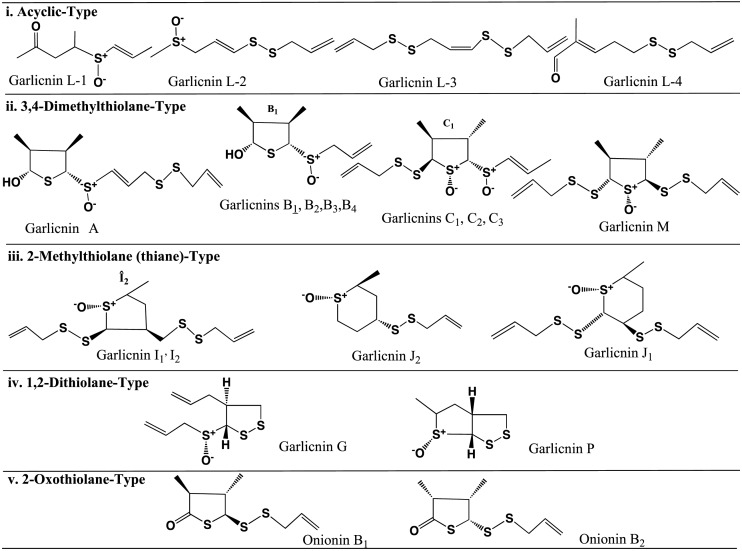
Structures of garlicnins and onionins isolated from garlic

## Hypothetic pathways to respective sulfides

**Fig. 4:**
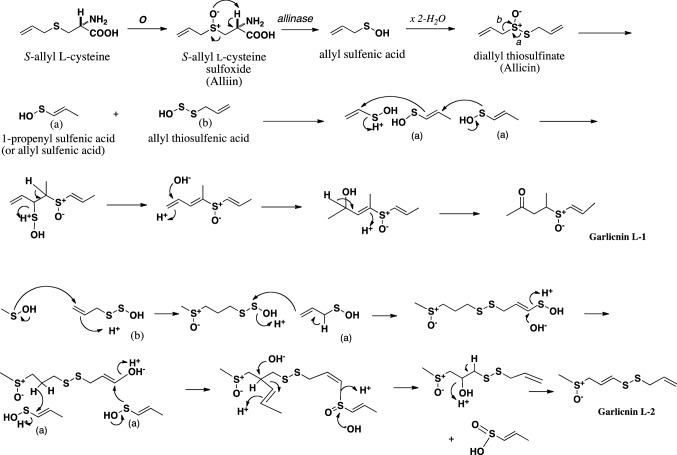
Hypothetical pathways to acyclic-type sulfides, garlicnins L-1 and L-2

**Fig. 5:**
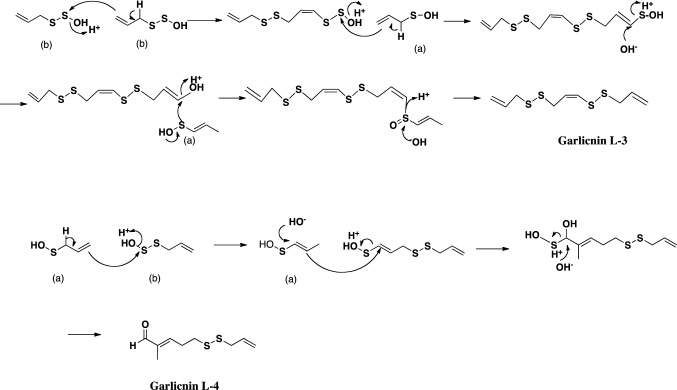
Hypothetical pathways to acyclic-type sulfides, garlicnins L-3 and L-4

**Fig. 6:**
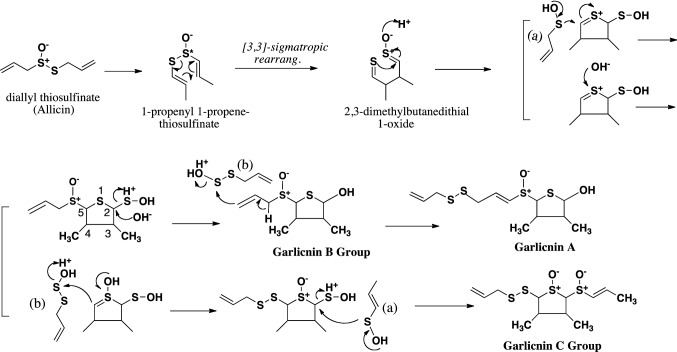
Hypothetical pathways to 3,4-dimethylthiolane-type sulfides, garlicnins A, B, and C groups

**Fig. 7:**
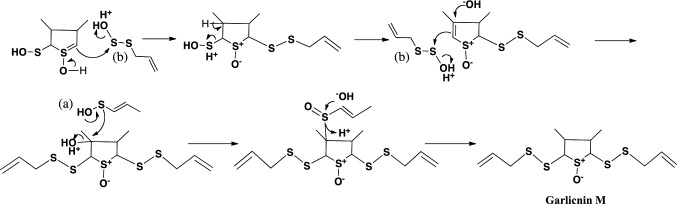
Hypothetical pathway to 3,4-dimethylthiolane-type sulfoxide, garlicnins M

**Fig. 8:**
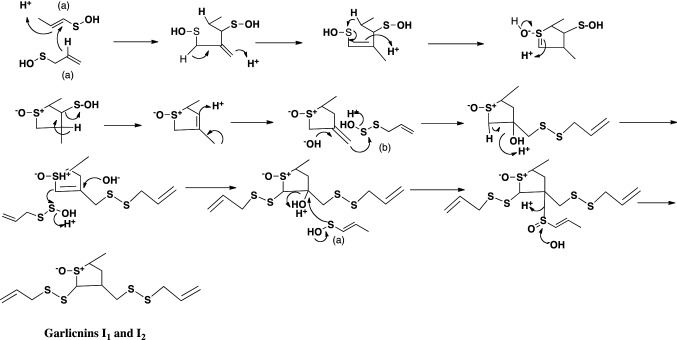
Hypothetical pathway to 2-methylthiolane-type sulfoxides, garlicnins I_1_ and I_2_

**Fig. 9:**
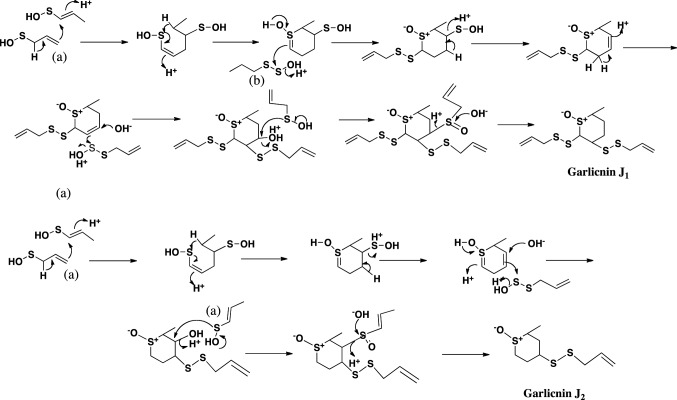
Hypothetical pathway to 2-methylthiane-type sulfoxides, garlicnins J_1_ and J_2_

**Fig. 10:**
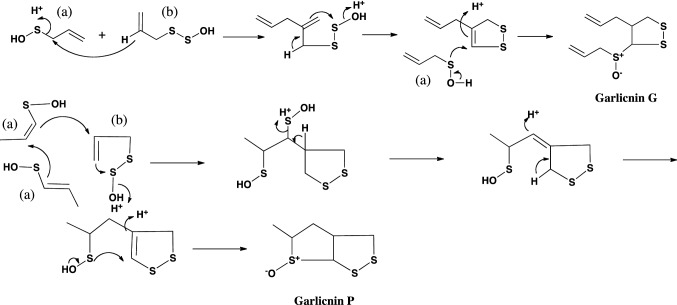
Hypothetical Pathway to 1,2-dithiolane-type sulfoxides, garlicnins G and P

**Fig. 11:**
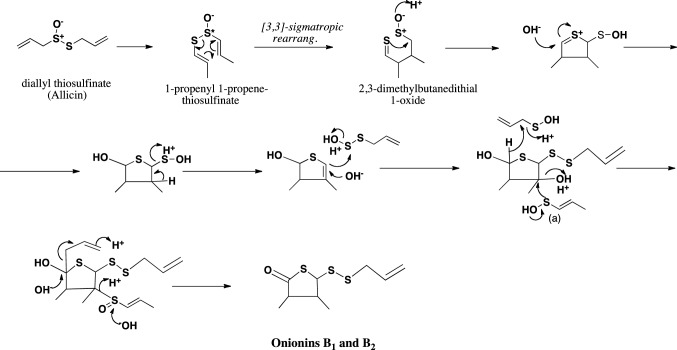
Hypothetical pathway to 2-oxothiolane-type sulfides, onionins B_1_ and B_2_

The first acyclic-type sulfides were produced by the arrangement and combination of allyl (or 1-propenyl) sulfenic acid, and allyl thiosulfenic acid derived from allicin (Fig. 4, Fig. 5). In the case of garlicnins L-1 and L-2, vinyl (ethenyl) and methyl sulfenic acid, respectively, were used in the first step of their synthesis. Moreover, on the basis of garlicnin L-2 formation, it was hypothesized that allyl (or 1-propenyl) sulfenic acid would be involved in hydroxylation for oxidative reaction, of which example were observed in the pathway to onionin B group. In the case of garlicnin L-2 formation, 1-propenyl sulfenic acid was likely involved in the dehydroxylation for reductive reaction, for which instances were observed in the pathways to garlicnins L-3, M, I_1_, I_2_, J_2_, and onionin B group. Formation of garlicnin B group was proposed as shown in Fig. 6: allicin was firstly derived from *S*-allyl L-cysteine, next allicin was transformed into 1-propenyl 1-propene-thiosulfinate via double-bond rearrangement and was then converted to 2,3-dimethylbutanedithial 1-oxide via [3,3]-sigmatropic rearrangement [30]. The generated intermediate was subsequently ring-closed to form a thiolane derivative that reacted with allyl sulfenic acid to finally produce the 3,4-dimethylthiolane-type sulfides, garlicnins B_1_, B_2_, B_3_ and B_4_. On the other hand, the above thiolane derivative was once hydroxylated on *S* in the thiolane framework to give thiolane *S*-oxide, and next reacted with allyl thiosulfenic acid and 1-propenyl sulfenic acid to generate the garlicnin C group, as shown in Fig. 6. The hypothetical pathway for the production of garlicnin M is shown in Fig. 7. In the production of the 2-methylthiolane(and thiane)-type sulfoxides, the combination of C-2 on allyl sulfenic acid and C-1 on 1-propenyl sulfenic was triggered in the pathways to garlicnins I_1_ and I_2_ as shown in Fig. 8, and the combination between the C-1 on 1-propenyl sulfenic acid and C-3 on allyl sulfenic acid occurred for the formation of garlicnin J_1_ as shown in Fig. 9. In the production of 1,2-dithiolane-type sulfoxides, the first stage was initiated by the combination of C-1 on allyl sulfenic acid and C-2 on allyl thiosulfenic acid in the case of garlicnin G. To produce garlicnin P, the dehydroxylation of allyl thiosulfenic acid resulted in successive rearrangements between allyl thiosulfenic acid and 1-propenyl sulfenic acid to yield garlicnin P as shown in Fig. 10. The 2-oxothiolane-type sulfides, onionins B_1_ and B_2_ were produced following hydroxylation to C-2 on the thiolane framework. This method differed from garlicnins C group, in which the hydroxylation to the *S atom* on the thiolane framework occurred as shown in Fig. 6. Furthermore, allyl sulfenic acid preferred hydroxylation at C-2 and 1-propenyl sulfenic acid may participate in dehydroxylation at C-4 as shown in Fig. 11.

## Effect of 3,4-dimethylthiolane-type sulfide [onionin A_1_ (1)] on tumor progression and metastasis in tumor injected mice

**Fig. 12:**
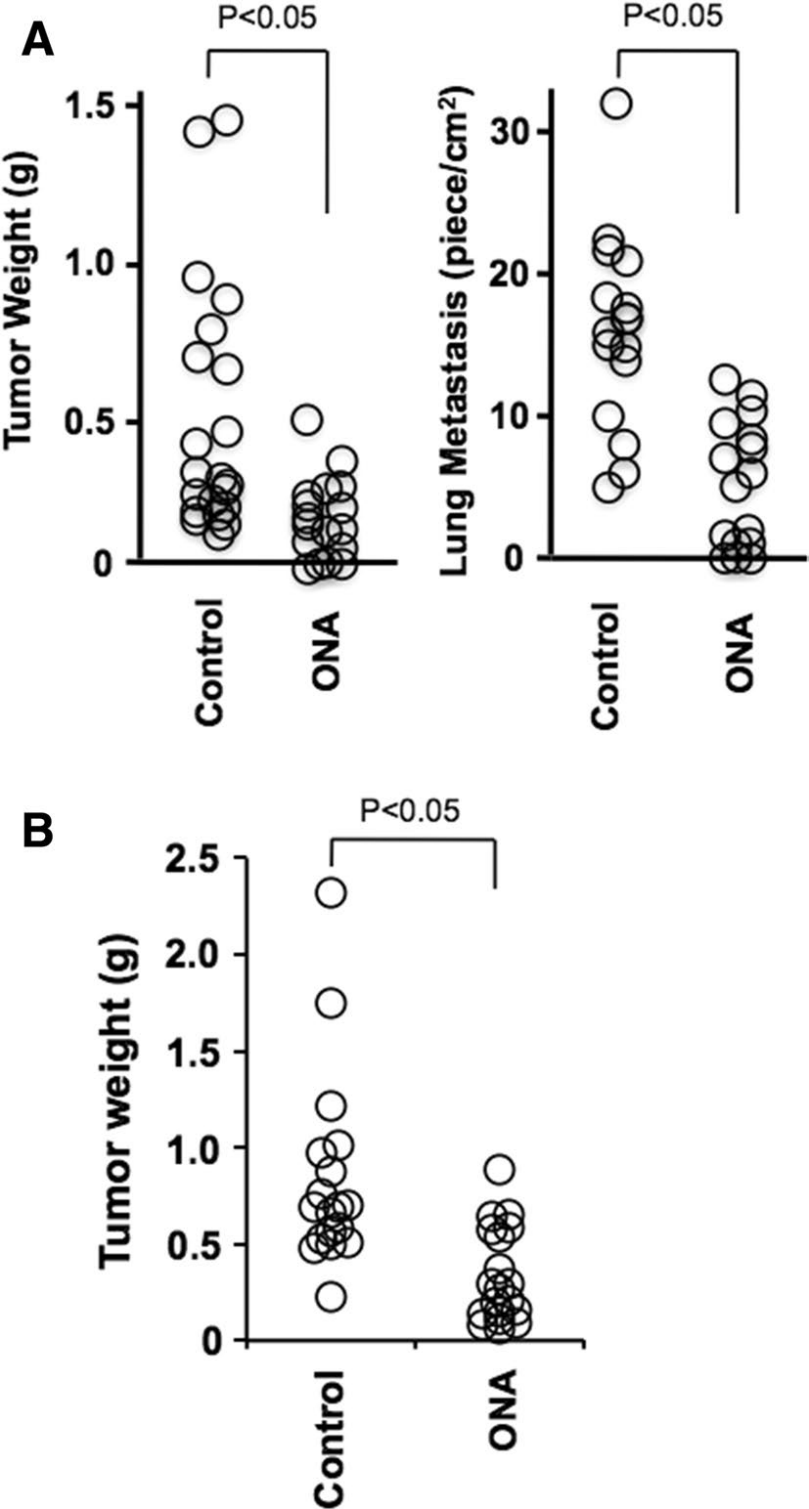
Effect of 3,4-Dimethylthiolane-type sulfide (onionin A_1_: ONA) on tumor progression and metastasis in tumor injected mice Onionin A_1_ (ONA) (20 mg/kg) was administered orally before and after the subcutaneous implantation of LM8 cells in the C3H mice (*n* = 20, each group) for 3 weeks, followed by determination of the subcutaneous tumor weight and presence of lung metastasis (**A**). As a murine ovarian cancer model, C57B6 mice were injected in the right ovary with iMOC cells and were administered ONA (20 mg/kg) for 3 week, followed by determination of the subcutaneous tumor weight (**B**)

3,4-Dimethylthiolane-type sulfides, such as onionins A_1_–A_3_ from onion and Welsh onion, and garlicnins A, B_1_–B_4_, C_1_–C_3_, and M from garlic are common compounds among these *Allium* species and are regarded as major sulfides. Therefore, to examine the antitumor activity, onionin A_1_ (**1**) [23], which is representative of the 3,4-dimethylthiolane-type sulfides, was investigated. Onionin A_1_ is an isomer of garlicnin B_1_, with an allylsulfinyl group instead of a 1-propenylsulfinyl group at C-5 on the core 3,4-dimethylthiolane 2-ol framework. Therefore, if onionin A_1_ is active for antitumor effects then garlicnin B_1_ also expected to be active. We used onionin A_1_ available at this time for antitumor examination. The effects of onionin A_1_ on tumor progression and metastasis in mouse osteosarcoma and ovarian cancer-bearing mouse models were investigated. Administration of onionin A_1_ significantly suppressed both subcutaneous tumor development and lung metastasis in a mouse osteosarcoma (LM-8)-bearing mouse model (**A** in Fig. 12). Furthermore, onionin A_1_ significantly suppressed (in promotion stage) tumor progression in a mouse ovarian cancer (iMOC)-bearing mouse model (**B** in Fig. 12), suggesting that onionin A_1_ is an orally available small molecule for anti-cancer therapy [31, 32]. The antitumor effects observed in vivo are likely caused by reversal of the antitumor immune system. Activation of the antitumor immune system by onionin A_1_ might be an effective adjuvant therapy for patients with osteosarcoma, ovarian cancer and other malignant tumors.

## Conclusion

**Fig. 13:**
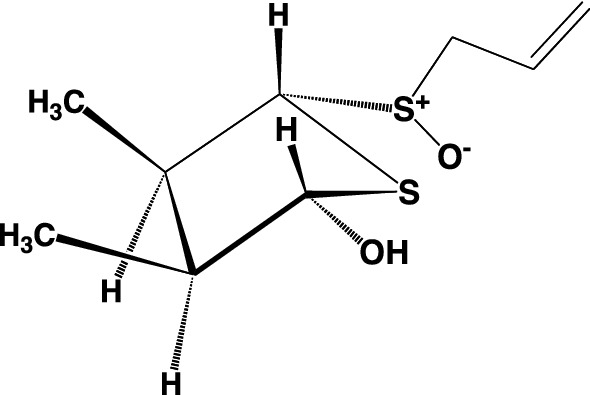
Garlicnin B_1_ (**2**)

The identification and characterization of novel sulfides isolated from garlic, onion, and Welsh onion have contributed to the identification of new chemicals and pharmaceutical compounds. Among the 3,4-dimethylthiolane-type of major sulfides, garlicnin B_1_ (Table 1, Fig. 13) is expected to be developed as a novel anti-cancer agent, as it is readily isolated in high yield, representing approximately 0.05% of Chinese garlic, and is also a synthesizable target because of its structural simplicity. Based on these findings, pharmacological investigations will be conducted to develop healthy foods and anti-cancer agents that can prevent or combat disease.
